# Empowering Communication in Medicine: A Workshop Approach to Improve Presentation Skills for Ear, Nose, and Throat Specialists

**DOI:** 10.7759/cureus.76342

**Published:** 2024-12-24

**Authors:** Takeshi Tsuda, Takumi Kumai, Yoshimasa Imoto, Michihisa Kono, Toshihiro Kishikawa, Kiyohito Hosokawa, Masafumi Sakashita

**Affiliations:** 1 Department of Otolaryngology - Head and Neck Surgery, Osaka University Graduate School of Medicine, Suita, JPN; 2 Department of Otolaryngology - Head and Neck Surgery, Asahikawa Medical University, Asahikawa, JPN; 3 Department of Otolaryngology - Head and Neck Surgery, University of Fukui, Yoshida, JPN

**Keywords:** communication training, elevator pitches, ent specialists, medical workshops, presentation skills, visual aids

## Abstract

Introduction

Effective communication is crucial for healthcare professionals, impacting patient care and interdisciplinary collaboration. However, medical education often lacks structured training in communication and presentation techniques. Herein, we evaluate the efficacy of structured workshops aimed at enhancing presentation skills among ear, nose, and throat (ENT) doctors through training in visual material design and concise verbal communication, including elevator pitches.

Methods

Two two-hour workshops were conducted for 23 ENT doctors under 50 years old, each with over 10 years of experience. The first workshop focused on creating effective visual aids, whereas the second targeted verbal communication skills. The questionnaire was administered before and after the workshops. Pre- and post-workshop questionnaires, using a 10-point Likert scale, evaluated participants' self-perceived abilities.

Results

The first workshop led to significant improvement in creating visually appealing and informative slides, using appropriate color schemes, and incorporating concrete examples. Despite a positive correlation with prior experience, time management did not improve significantly. The second workshop revealed significant gains in verbal communication skills, including concise messaging, audience engagement, and the effective use of storytelling techniques. Participants improved their ability to streamline information, maintain a conversational tone, and use eye contact and gestures effectively.

Conclusion

This study demonstrated that structured workshops significantly improved the presentation skills of ENT doctors, particularly in visual material design and verbal communication. Integrating such training into medical education and professional development can enhance patient care and interdisciplinary collaboration by fostering clear, effective communication among healthcare providers.

## Introduction

Effective communication is a critical skill for healthcare professionals, as the precise and clear exchange of information can profoundly influence patient care and interdisciplinary collaboration. Presenting complex medical concepts in a concise and engaging manner is not only vital for educating clinicians and patients, but also essential for enhancing overall healthcare delivery. However, despite its importance, structured training in communication and presentation techniques is often underrepresented in medical education. Recent initiatives to bridge this gap have focused on targeted interventions, such as workshops and hands-on sessions, designed to develop both visual and verbal communication skills [1]. Research has demonstrated that improving these abilities during residency or early-career stages enhances healthcare providers’ confidence and supports their professional growth. These advancements contribute to more effective patient interactions and more informed clinical decision-making [2].

Workshops emphasizing the creation of effective visual aids and clear articulation of key points, such as elevator pitches, have proven to significantly enhance communication efficiency among medical professionals. This is particularly crucial in clinical settings, where time constraints often necessitate the rapid and accurate dissemination of critical information. Research has demonstrated that healthcare providers who receive structured training in presentation skills show significant improvements in their ability to organize and present data, thereby enhancing mutual understanding and collaboration among interdisciplinary teams [3]. These findings highlight the value of integrating presentation skills training into medical curricula, ultimately contributing to improved healthcare outcomes.

Although structured communication training is increasingly incorporated into early medical education, few studies have specifically targeted ear, nose, and throat (ENT) specialists [4-6]. In this study, we aim to evaluate whether structured workshops on visual material design and verbal communication can enhance the presentation skills of ENT professionals.

## Materials and methods

Workshop contents

The workshop was divided into two sessions, each lasting approximately two hours. Both sessions included a lecture, followed by small group activities and presentations to the entire group.

The first session focused on designing effective presentation materials using presentation software (e.g., PowerPoint®; Microsoft® Corp., Redmond, WA, USA). The group work focused on two themes: first, creating a clear slide presentation to explain the pathogenesis of a particular disease; second, preparing a similar presentation to highlight the importance and treatment of a specific disease.

The second session emphasized verbal communication skills, particularly the delivery of concise presentations, such as elevator pitches. Group work was conducted on two themes: delivering a 30-second elevator pitch about the participants' current work focus, and a 30-second explanation of the drug to a patient, assuming informed consent. Both sessions featured interactive activities, practical exercises, and peer feedback to consolidate learning. Surveys were administered to participants before and after each workshop session to assess changes in their self-perceived presentation abilities.

Participants

As mentioned earlier, both workshops utilized role-playing activities conducted in small groups. Consequently, the sample size was determined based on feasibility and availability, and a pilot study design was employed. The inclusion criteria were ENT doctors from across Japan and doctors under the age of 50. The exclusion criteria included doctors holding positions as professors or associate professors, and those unable to attend in person.

As a result, 23 ENT doctors, recruited from various healthcare institutions in Japan, participated in this study. All physicians had been practicing medicine for over 10 years. Twelve ENT doctors attended the first workshop, and 12 questionnaires were collected. Twenty-three doctors participated in the second workshop, and 20 questionnaires were retrieved. Of these, one doctor attended only the first workshop, 11 attended only the second, and 11 attended both workshops. At our institution, questionnaires conducted as part of the survey that do not involve health promotion or similar activities do not require review board approval. This study complied with the Personal Information Protection Law, and all data were provided in a fully anonymized format, ensuring that there were no direct or indirect links to individual identities. The data were received and analyzed under these conditions.

Data collection

The first questionnaire was administered both before and after the first workshop to evaluate participants' self-perceived ability to design effective visual aids, as outlined in Table 1.

**Table 1:** Workshop questionnaire used for the first session

Question Number	Evaluation Criteria	Description/Assessment Focus
Question 1	Accuracy of Content	Is the content of the presentation based on accurate and reliable information?
Question 2	Clarity of Topic	Is the subject matter and purpose of the presentation clearly related?
Question 3	Organization of Information	Is the information organized in a logical and effective manner?
Question 4	Emphasis of Importance	Are key points and important information appropriately emphasized?
Question 5	Visual Appeal	Is the design of the slides visually appealing? Are color and font choices appropriate?
Question 6	Ease of Understanding	Is the presentation content easy for the audience to understand?
Question 7	Interactivity	Does the presentation include opportunities for audience interaction and questions?
Question 8	Use of Concrete Examples	Are examples and data used to illustrate the presentation?
Question 9	Overall Flow	Does the presentation flow smoothly, with natural transitions?
Question 10	Time Management	Is the presentation properly completed within the allotted time?

This questionnaire focused on assessing skills related to the preparation of presentation materials. The second questionnaire, conducted before and after the second workshop, evaluated presentation techniques with an emphasis on elevator pitches. The detailed contents of the second questionnaire are shown in Table 2.

**Table 2:** Workshop questionnaire used for the second session

Question Number	Evaluation Criteria	Description/Assessment Focus
Question 1	Concise Communication	Can you convey your opinion concisely in any situation?
Question 2	Conclusion	Can you effectively convey the conclusion you want to make?
Question 3	Audience's Perspective	Do you structure your presentation from the audience's point of view?
Question 4	Ability to Capture Interest	Can you capture the interest of your audience in a short period of time?
Question 5	Elimination of Unnecessary Information	Can you focus on the main points by omitting unnecessary details?
Question 6	Natural Conversation	Do you speak in a natural manner?
Question 7	Tone and Speed of Voice	Can you adjust your tone and speed to match the content?
Question 8	Getting to the Point Promptly	Can you reach key messages within the allotted time?
Question 9	Eye Contact and Gestures	Can you make eye contact with the audience and use gestures appropriately?
Question 10	Use of Storytelling	Are you mindful of the content flow during your presentation?

Both questionnaires utilized a 10-point Likert scale, where 1 indicated low proficiency and 10 indicated high proficiency.

Statistical analysis

All statistical analyses were conducted using Prism, version 7 (GraphPad Software, San Diego, CA, USA). Data are presented as means ± standard deviation. Comparisons between the pre- and post-workshop scores were performed using the nonparametric Wilcoxon test. Spearman’s rank correlation coefficient was employed to assess correlations between variables. Statistical significance was defined as p < 0.05.

## Results

First workshop (preparation of presentation materials)

**Figure 1:**
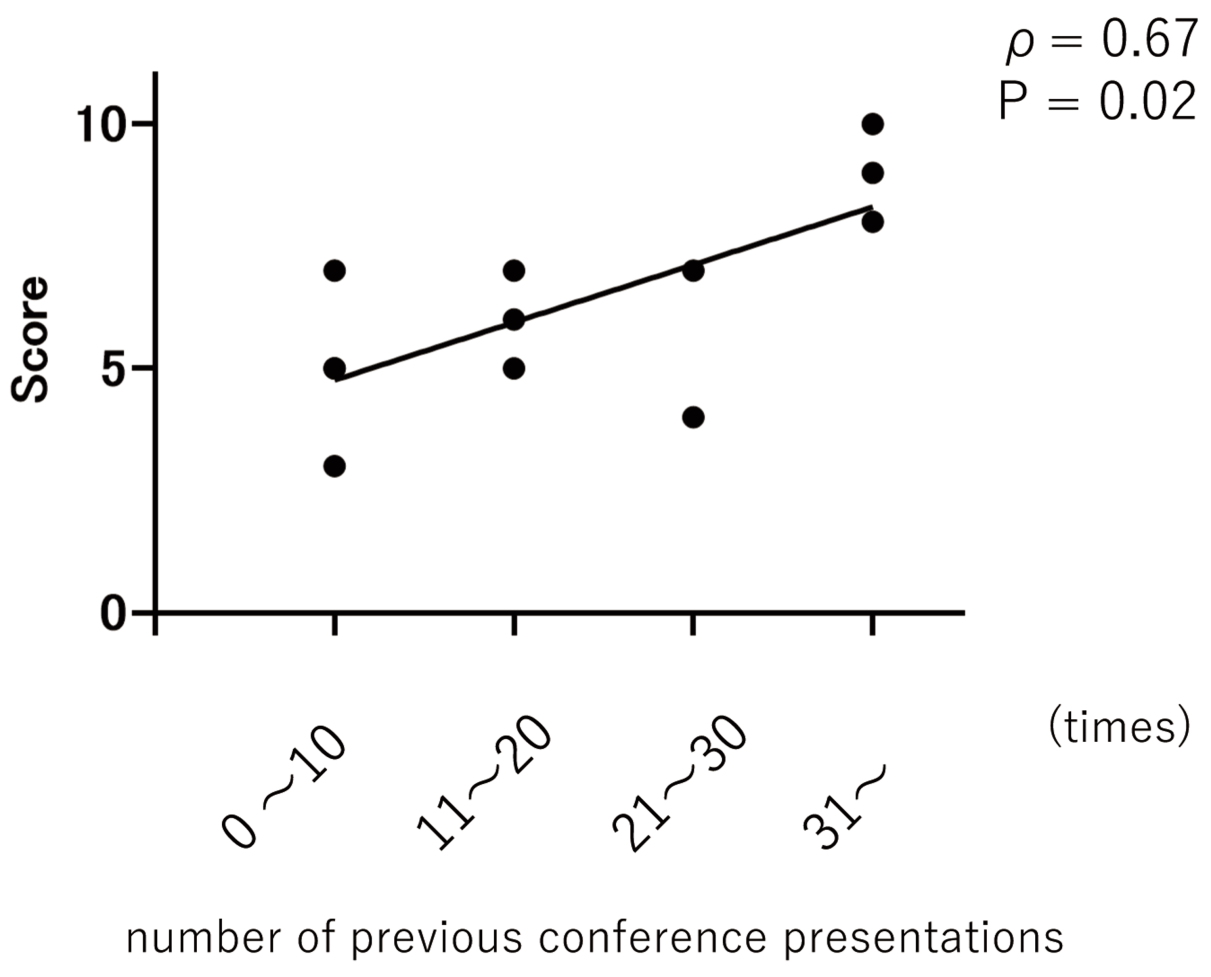
Correlation between the number of previous conference presentations and self-evaluation using specific examples A significant positive correlation was identified between the number of previous conference presentations and participants’ self-assessment scores for the use of specific, appropriate examples.

The first workshop assessed participants on 10 aspects related to the preparation of presentation materials, each rated on a 10-point scale. A pre-workshop survey revealed a positive correlation between the use of concrete examples and the number of previous conference presentations, suggesting that participants with more experience were more proficient in incorporating concrete examples (Spearman’s rank correlation coefficient, ρ = 0.67, p = 0.02) (Figure 1).

**Figure 2:**
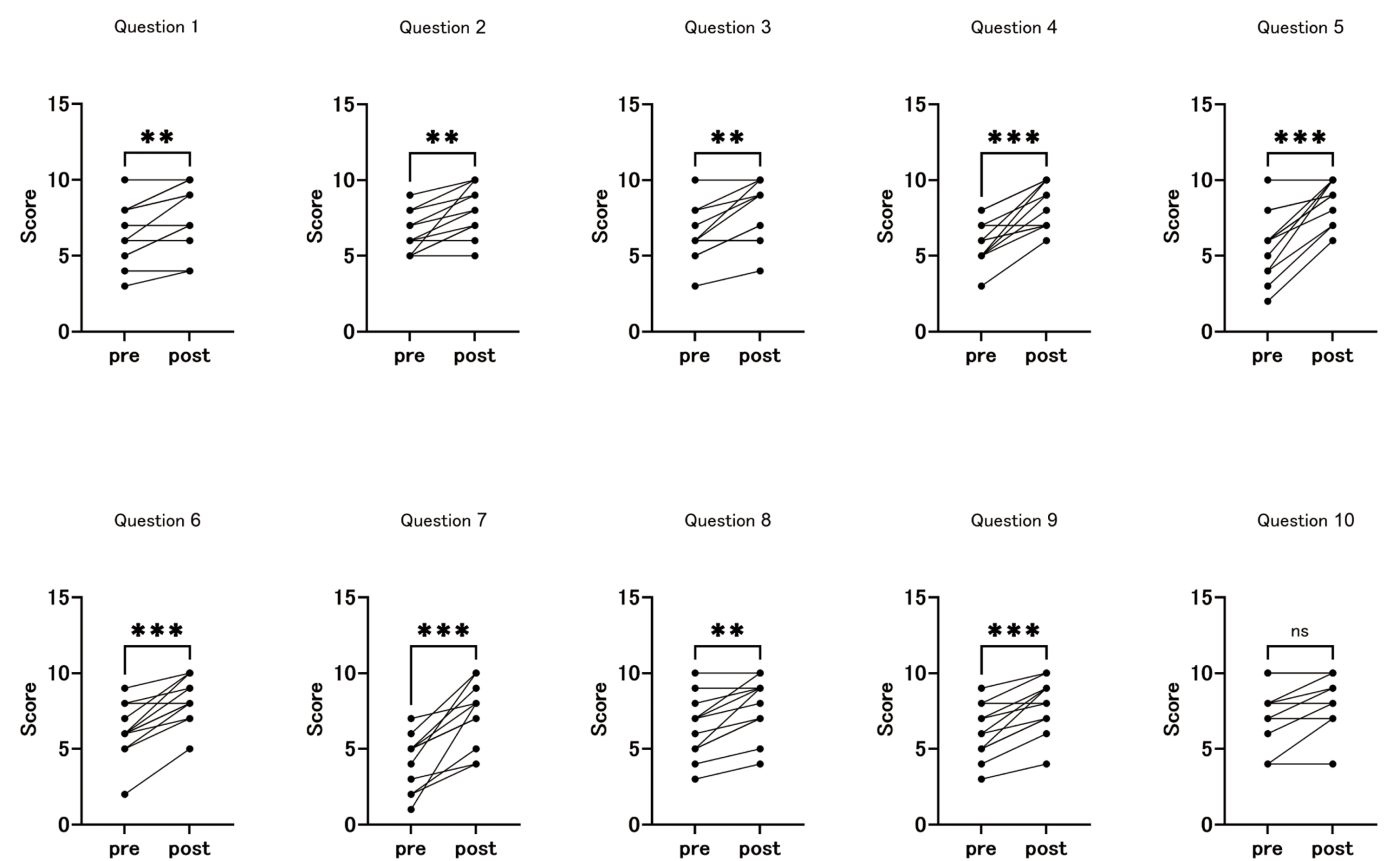
Pre- and post-workshop self-assessment by questionnaires (first workshop) All assessed items, except time management, showed significant improvements in self-rated scores from pre- to post-workshop evaluations. **p < 0.01, ***p < 0.001, ns: not significant (Wilcoxon test)

Post-workshop analysis indicated significant improvements in nearly all assessed areas, except for time management (Figure 2).

Participants demonstrated marked progress in their ability to prepare presentations containing accurate and reliable information (pre-workshop: 6.5 ± 2.0; post-workshop: 7.7 ± 2.2, p = 0.0003). Moreover, they conveyed the subject matter and purpose of their presentations more effectively (pre-workshop: 6.4 ± 1.4; post-workshop: 8.0 ± 1.7, p = 0.0009). In terms of visual design, participants reported significant enhancements in slide aesthetics, including the use of appropriate colors and fonts to improve visual appeal. The organization of information improved significantly (pre-workshop: 6.3 ± 1.8; post-workshop: 7.8 ± 2.0, p = 0.0035), and participants made notable progress in emphasizing key points and important information (pre-workshop: 5.9 ± 1.4; post-workshop: 8.3 ± 1.5, p = 0.0005). Furthermore, the ease of understanding improved significantly, as participants demonstrated a greater ability to make content more comprehensible to the audience. Interactivity also increased, with participants fostering audience engagement through questions and discussions. Participants made notable progress in using concrete examples and data to illustrate key points, reflecting a more practical and impactful presentation style. The presentation flow also improved significantly, with smooth transitions between points. Time management skills remained relatively stable, with scores before the workshop (pre-workshop: 7.3 ± 1.9) and after (post-workshop: 8.2 ± 1.7) showing minimal change. However, time management positively correlated with the number of previous conference presentations, suggesting that more experienced participants were better at managing their time effectively during presentations. The lack of improvement in time management may indicate that foundational skills, such as time allocation, are more influenced by experience than by short-term training. Several participants reported that the skills gained during this session were immediately useful in improving the quality of presentations delivered at clinical meetings and academic conferences.

Second workshop (presentation techniques)

**Figure 3:**
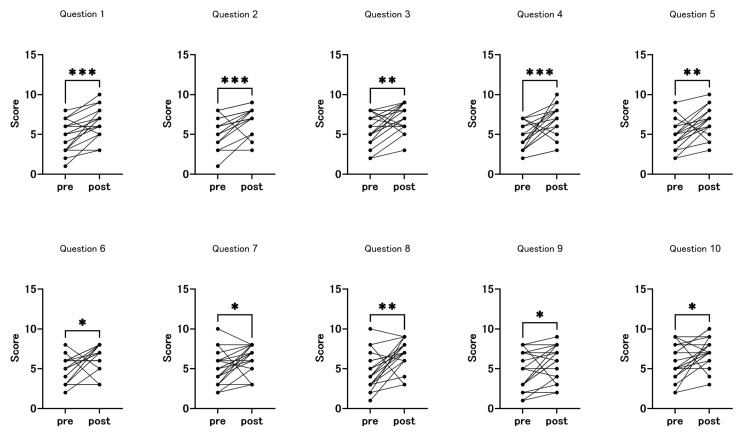
Pre- and post-workshop self-assessment by questionnaires (second workshop) All assessed items showed significant improvements in self-rated scores after the second workshop. *p < 0.05, **p < 0.01, ***p < 0.001, ns: not significant (Wilcoxon test)

The second workshop emphasized verbal communication skills and effective presentation techniques, focusing on concise communication, audience engagement, and impactful presentations within a limited timeframe. Significant improvements were observed across all evaluated items (Figure 3).

Participants demonstrated enhanced abilities to convey ideas concisely in various situations and to effectively communicate conclusions. There was a substantial improvement in structuring presentations from the audience's perspective, enabling participants to better consider and engage with their audience’s needs. They exhibited greater skill in capturing audience interest early and drawing attention effectively at the beginning of presentations. Statistical analysis revealed that the mean score for “Concise Communication” improved from 4.7 ± 1.9 (pre-workshop) to 6.5 ± 1.8 (post-workshop), and the “Audience's Perspective” also improved from 5.5 ± 1.9 to 7.1 ± 1.7, whereas the “Ability to Capture Interest” increased from 4.4 ± 1.7 to 6.7 ± 1.9 (p < 0.01). Similarly, the “Elimination of Unnecessary Information” improved from 4.8 ± 1.9 to 6.7 ± 1.8 (p = 0.0016), highlighting significant advancements in participants' ability to eliminate unnecessary information and focus on key points. They reported improvements in maintaining a conversational tone, with better control over tone and speed to align with the content. Participants also showed greater efficiency in reaching key points within the allotted time, reflecting significant advancement in concise communication (“Tone and Speed of Voice”: from 5.1 ± 2.1 to 6.4 ± 1.8, p = 0.016; “Getting to the Point Promptly”: from 4.8 ± 2.3 to 6.9 ± 1.8, p = 0.0019). Participants used eye contact, and gestures were used more effectively, contributing to a more engaging delivery. They also demonstrated greater awareness of narrative flow, employing storytelling techniques to logically guide the audience through the content. Feedback collected informally after the workshop indicated that participants felt more confident presenting research findings and clinical case summaries during team meetings and professional conferences.

## Discussion

This study demonstrates the effectiveness of structured workshops in improving presentation skills among ENT professionals, with significant enhancement observed in both the design of visual materials and verbal communication techniques. Our findings are consistent with prior research, which highlights persistent gaps in presentation skills among healthcare professionals due to insufficient training [7-10]. Clarke et al. reported that healthcare providers frequently rely on bullet points and text-heavy slides, often expressing the least confidence in storytelling [7]. By addressing these gaps, our workshops focused on the strategic use of visual aids and storytelling techniques, equipping participants to create visually appealing and engaging presentations. This approach successfully overcame common barriers to effective communication, enabling participants to deliver presentations with greater impact.

In contrast to Watts et al. [11], who identified time management as a significant challenge for surgical trainees, our study found that time management was largely manageable among participants, with a median pre-workshop score of 8. This discrepancy is likely attributable to the extensive experience of our participants, most of whom had over a decade of medical practice and considerable experience presenting at academic conferences. These findings suggest that, while foundational presentation skills - including time management - may already be well developed among experienced physicians, structured workshops can still play a pivotal role in refining other critical aspects, such as content clarity and audience engagement.

The workshops also significantly enhanced participants' verbal communication skills, particularly through the application of elevator pitch techniques. This aligns with the findings of Hicks [12], who emphasized the utility of elevator pitches as networking tools for healthcare professionals, enabling succinct introductions that highlight key topics in under 150 words. Similarly, Wankah et al. highlighted the value of elevator pitches for early-career researchers, allowing them to quickly capture stakeholders’ interests in complex topics [13]. These skills were further reinforced by Kirchoff’s advocacy for the “and-but-therefore” format, which promotes concise and impactful presentations [14]. Collectively, these findings underscore that structured training empowers healthcare professionals with persuasive communication abilities, benefiting their professional interactions.

Additionally, significant improvements in soft skills, such as audience engagement and concise communication, align with Snigdha et al.’s findings, which focused on training interventions that substantially enhanced both content quality and soft skills among ophthalmology fellows [15]. Participants in our study similarly demonstrated gains in clarity, audience connection, and storytelling abilities - all essential for impactful communication in clinical and interdisciplinary settings. These results suggest that hands-on, interactive training can effectively address presentation skill gaps, fostering confidence and professionalism among healthcare providers, which may, in turn, improve patient care and interdisciplinary collaboration.

This study has some limitations that should be addressed. First, the reliance on self-assessment data introduces biases, such as the potential overestimation or underestimation of participants' abilities, due to its inherently subjective nature. Future studies could address this limitation by incorporating objective measures, such as independent evaluations of presentations conducted by trained assessors or real-world performance assessments at conferences or clinical meetings. Second, the small sample size (less than 30 participants) in this pilot study limits the generalizability of our findings. Large-scale studies with more diverse participants are pertinent to confirm these trends and strengthen the reliability of our results. Finally, we assessed only short-term improvements in presentation skills immediately after the workshops. Although participants provided positive feedback, this feedback was subjective and lacked quantitative evaluation. The long-term retention and application of these skills in professional practice remain unknown. Future workshops could address this limitation by incorporating follow-up video-recorded assessments conducted several months after the intervention. Such evaluations would allow for a more objective analysis of skill retention and the extent to which participants apply these skills over time.

Despite these limitations, our findings highlight the significant practical relevance of structured communication workshops in clinical and interdisciplinary settings. Enhanced presentation and communication skills can improve patient care by enabling physicians to convey complex medical information clearly and concisely. For instance, effectively explaining diagnoses, treatment plans, or surgical procedures can foster greater understanding and trust, leading to better-informed decisions and adherence to care plans. Additionally, enhanced communication improves interdisciplinary teamwork by ensuring the efficient exchange of critical information during case discussions, surgical planning, or emergency situations. Clarity and conciseness are particularly valuable in time-sensitive scenarios, where accurate information sharing is essential for patient safety and optimal clinical outcomes. By equipping healthcare professionals with these skills, structured workshops like ours contribute to improved patient care, stronger interdisciplinary collaboration, and enhanced professional communication.

## Conclusions

This study evaluates the effectiveness of structured workshops in enhancing the presentation skills of ENT professionals by emphasizing visual material design and concise verbal communication, using elevator pitches. The findings demonstrate that the training sessions significantly improved participants’ abilities to create accurate, visually appealing presentations and deliver concise messages.

These results support the integration of communication training into medical education and professional development programs, highlighting the importance of clear and effective communication in clinical and interdisciplinary contexts. We recommend the integration of structured communication workshops into comprehensive medical education curricula to enhance presentation and communication skills across all medical specialties. Future studies should expand on these findings by validating their impact in larger cohorts or exploring their applicability across different medical fields, ultimately reinforcing communication competencies as core skills that enhance patient care and interdisciplinary collaboration through improved presentations.
